# Aberrant expression of CD133 in non-small cell lung cancer and its relationship to vasculogenic mimicry

**DOI:** 10.1186/1471-2407-12-535

**Published:** 2012-11-21

**Authors:** Shiwu Wu, Lan Yu, Danna Wang, Lei Zhou, Zenong Cheng, Damin Chai, Li Ma, Yisheng Tao

**Affiliations:** 1 Department of Pathology, the First Affiliated Hospital of Bengbu Medical College, Bengbu Medical College, Bengbu 233000, Anhui Province, China

## Abstract

**Background:**

To investigate on expressions and clinical significances of CD133 protein and vasculogenic mimicry (VM) in primary non-small cell lung cancer (NSCLC).

**Methods:**

The specimens of NSCLC from 305 Chinese patients with follow-up were analyzed for CD133 protein expression and VM by immunohistochemical and histochemical staining.

**Results:**

In NSCLC, positive rates of 48.9% and 35.7% were obtained for CD133 and VM, respectively. The VM and expression of CD133 were significantly higher in carcinoma than in normal. There were a positive relationship between the VM and expression of CD133 and the tumor grade, lymph node metastasis and clinical stage (all P<0.05). The overall mean survival time of the patients with CD133 and VM positive expression was lower than that of patients with negative expression. Microvessel density (MVD) was positive corresponded with the grade, lymph node metastasis and clinical stage (all P<0.05). The overall mean survival time of the patients with MVD≥22’s group was shorter than that of patients with MVD<22’s group. Pathological-tumor-node-metastasis (pTNM) stage, positive expression of CD133 and VM, postoperative therapy and MVD were independent prognostic factors of NSCLC (P<0.05). Immunohistochemistry revealed an important intratumoral heterogeneity in all four CD133 expression profiles.

**Conclusions:**

VM, MVD and expression of CD133 are related to differentiation, lymph node metastasis, clinical stage, and prognosis. It is suggested that CD133, VM and MVD should be considered as a potential marker for the prognosis.

## Background

Lung cancer is among the most common form of cancer of the respiratory system with an estimated incidence of approximately 220,000 cases in the USA [1], and is the leading cause of cancer-related mortality worldwide, with nearly 1.4 million deaths each year [2], and non-small cell lung cancer (NSCLC) accounts for nearly 85% of all cases of lung cancer [1]. Worldwide, NSCLC is the most common form of cancer with overall 5-year survival rate of less than 20.0% as most patients are diagnosed late and are unsuitable for curative surgery. A major problem in finding treatments is the frequent resistance to drugs which emerges. This is linked to the development and maintenance of small population of tumor cells, termed cancer stem cells (CSCs) or tumor-initiating cells (TIC). These cells have been implicated in self-renewal, progression, and therapy-resistance in multiple solid cancers and high tumorigenicity [3-13]. A commonly CSCs marker is CD133, also known as prominin-1, a 120 kDa five transmembrane domain cell surface glycoprotein, is originally described as a surface antigen specific for human hematopoietic stem and progenitor cells [14,15]. CD133 is overexpressed in various solid tumors, including brain [16], endometrial cancer [17], kidney [18], lung [19], liver [20], gastric [21,22], colon [23], pancreas [24], breast [25], skin [26] and prostate [27].

About tumor blood supply, the traditionally much attention has been focused on the role of angiogenesis [28]. Recently, Maniotis [29] and his coworkers had described an angiogenesis-independent pathway called “vasculogenic mimicry (VM)”, a new phenomenon in which highly aggressive human melanoma cell mimic endothelial cells and form vascular channel-like structures to convey blood without the participation of endothelial cells. VM consists of three formations: the plasticity of malignant tumor cells, remodeling of the extracellular matrix (ECM), and the connection of the VM channels to the host microcirculation system [30-33]. Later, in many aggressive tumors, including breast carcinoma [34], glioma [35], hepatocellular carcinoma [36], clear cell renal cell carcinoma [37], laryngeal squamous cell carcinoma [38], ovarian carcinoma [39], gastric adenocarcinoma [40] and prostate carcinoma [41], have been described. VM is associated with poor prognosis in tumor patients. The relationship between CD133 and VM in NSCLC has not yet been explored. In this study, we performed an immunohistochemical investigation to explore the role of the CD133 and VM in chinicopathology and prognosis in 305 cases of NSCLC.

## Methods

### Biopsy specimens

Paraffin embedded sections of 305 NSCLCs and 30 distal normal lung tissues were obtained from the Department of Pathology, the First Hospital Affiliated to Bengbu Medical College between 2003 and 2006 (Patients who had received preoperative chemotherapy or radiotherapy were excluded). This study was approved by the ethical committee of the First Hospital Affiliated to Bengbu Medical College before its start. The age of the patients ranged from 26–82 years, the median age was 59.8 years. Two hundred thirty-three were males and seventy-two were females. Among the 305 cases, 248 were to postoperative therapy (routine chemotherapy or radiotherapy). Thirty-four were at grade I, two hundred three at grade II, sixty-eight were at grade III, according to the grading system of the World Health Organization (WHO). Two hundred ten were squamous cell carcinoma and ninety-five were adenocarcinoma. Two hundred twenty-four were central type and eighty-one were peripheral type. From lymph node metastasis, consisted of one hundred twenty specimens of no metastasis and one hundred eighty-five of yes metastasis. Fifty were stage I, ninety were stage II, ninety-four were stage III and seventy-one were stage IV, according to clinical staging of pTNM, respectively.

### Immunohistochemistry

All samples were fixed 10% buffered formalin and embedded in paraffin. 4 μm thick tissue sections were cut. All sections were deparaffinnized and dehydrated with graded alcohol. The sections were then washed 10 min in phosphate-buffered saline (PBS) (pH 7.2). The endogenous peroxidase activity was quenched by incubation in methanol containing 3% H2O2 for 10 min at room temperature, then heated for 30 min at 95°C to repair antigens and finally rinsed in PBS. After several washes in PBS, the sections were blocked with goat serum for 20 min at room temperature, and then incubated with mouse monoclonal CD133 (Santa Cruz) and CD34 (LabVision) primary antibodies overnight at 4°C in a humidified chamber. Replacing the primary antibodies by PBS, negative control staining was using performed, the slides were treated with polymer enhancer (Reagent A) for 20 min at room temperature. After a complete wash in PBS, the slides were treated with goat anti-mouse antibody (Reagent B) for 30 min at room temperature. After a complete wash in PBS, the slides were develop in freshly prepared diaminobenzedine solution (DAB,) for 8 min, and then counterstained with hematoxylin, dehydrated, airdried, and mounted.

### Evaluation of score

Slides were reviewed independently by two observers to evaluate the staining pattern of the protein separately under the light microscopy. In scoring expression of CD133 and CD34 protein, both the extent and intensity of immunopositivity were considered. The intensity of positivity was scored as follows: 0, negative; 1, weak; 2, moderate; 3, strong. The extent of positivity was scored according to the percentage of cells showing positive staining: <10% is 1; 11%-50% is 2; 51%-75% is 3; >75% is 4. The final score was determined by multiplying the intensity of positivity and the extent of positivity scores, yielding a range from 0 to 12. The expression for CD133 and CD34 was considered positive when the scores were >1.

The staining for CD133 was mainly confined to the cytoplasm and membrane. The staining for CD34 was mainly confined to the cytoplasm and membrane and presented as brown granular materials. Microvessel density (MVD) was determined by the mean number of small CD34-positive vessels counted. A modified Weidner’s method was used to calculate the MVD of NSCLC by anti-CD34 immunostaining [42]. All samples were submitted for PAS-CD34 dual staining to characterize endothelial cells glycosylated basement membranes of vessels as well as vessel-like (VM) channels [43]. Moreover, no necrosis or hemorrhage in tumor tissue near the VM channels was noted. The technique was adopted from Yue et al. with some modifications [44]. The observers selected ten high power fields and counted individual microvessels.

### Statistical analysis

Fisher’s exact test, Pearson Chi-square test for trends in proportions, Spearman’s correlation coefficient test, and Kaplan-Meier’s method with log rank test or Cox Regression method for univariate or multivariate overall survival analysis were used to assess the associations among the positive staining of CD133 or VM or MVD and clinicopathological indices by SPSS 17.0 software for windows (Chicago, IL). A value of P<0.05 was recognized as statistically significant.

## Results

### The association between expression of CD133, VM and MVD and clinicopathological factors

**Figure 1:**
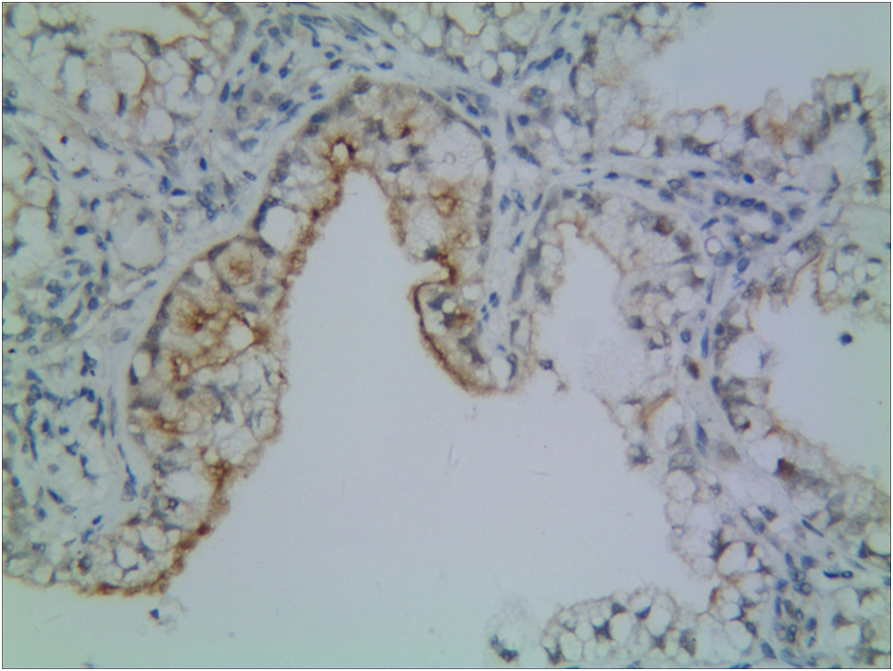
**Representative results of CD133 protein in non-small cell lung cancer and control group.** Control bronchiolar epithelial cells expressed CD133 in the membrane and cytoplasm.

**Figure 2:**
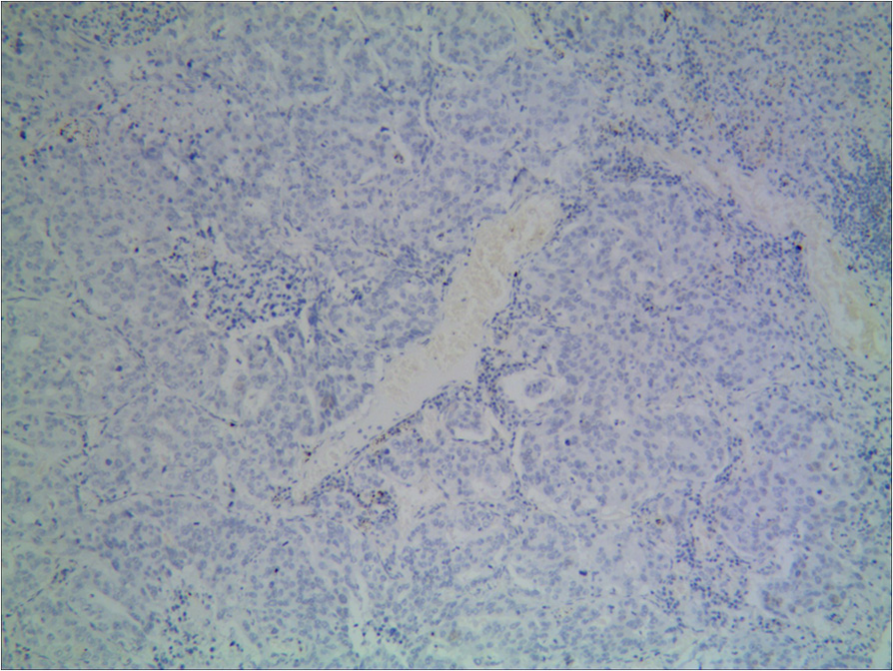
**Representative results of CD133 protein in non-small cell lung cancer and control group.** NSCLC cells did not express a detectable level of CD133.

**Figure 3:**
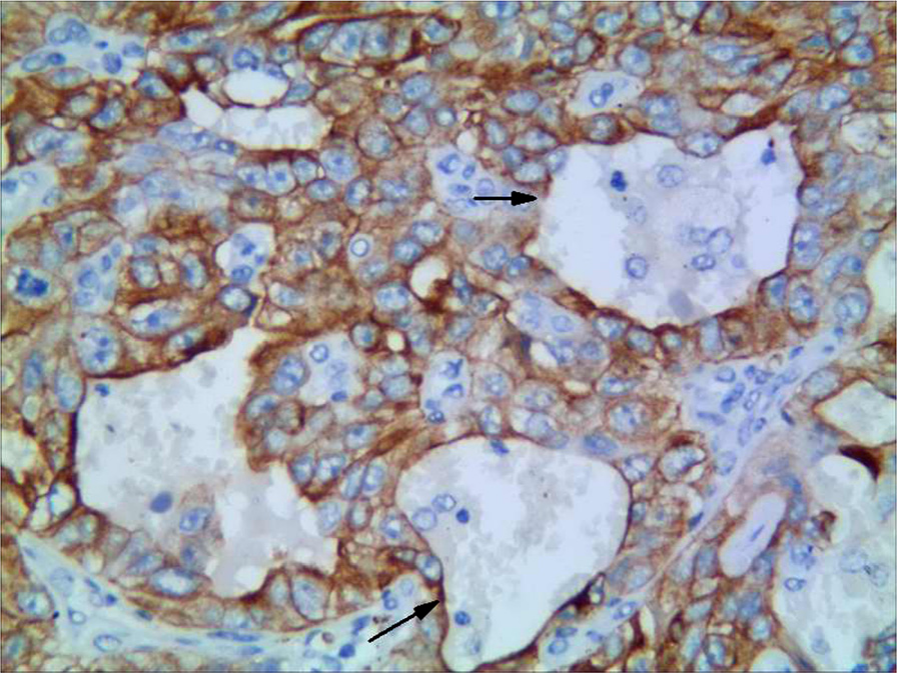
**Representative results of CD133 protein in non-small cell lung cancer and control group.** CD133 predominantly localized in the membrane and cytoplasm in moderately differentiated squamous cell carcinoma (grade 2) (CD133×400)(black arrow is VM).

**Figure 4:**
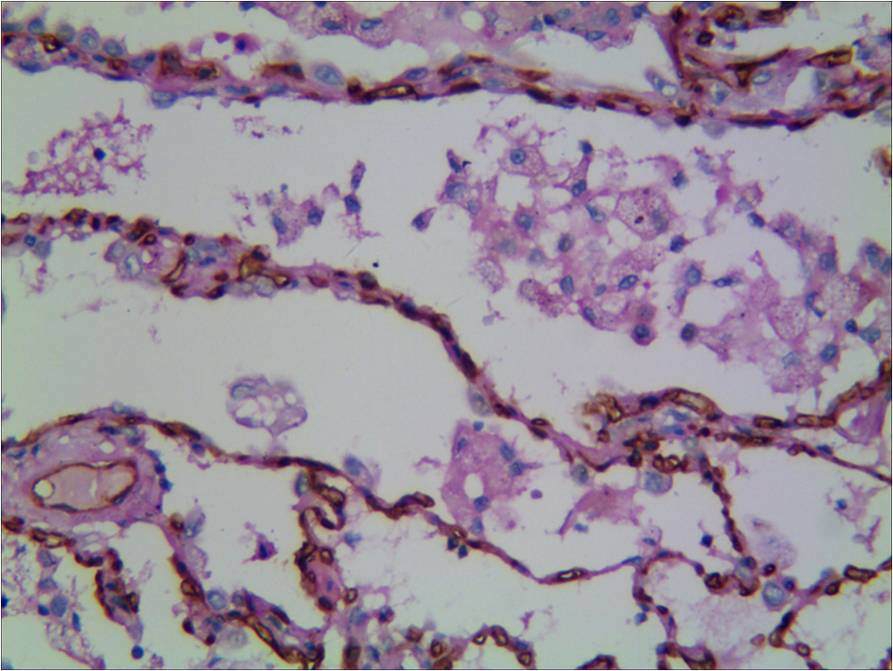
**Representative results of VM in non-small cell lung cancer and control group.** No VM’s phenomenon in the control group.

**Figure 5:**
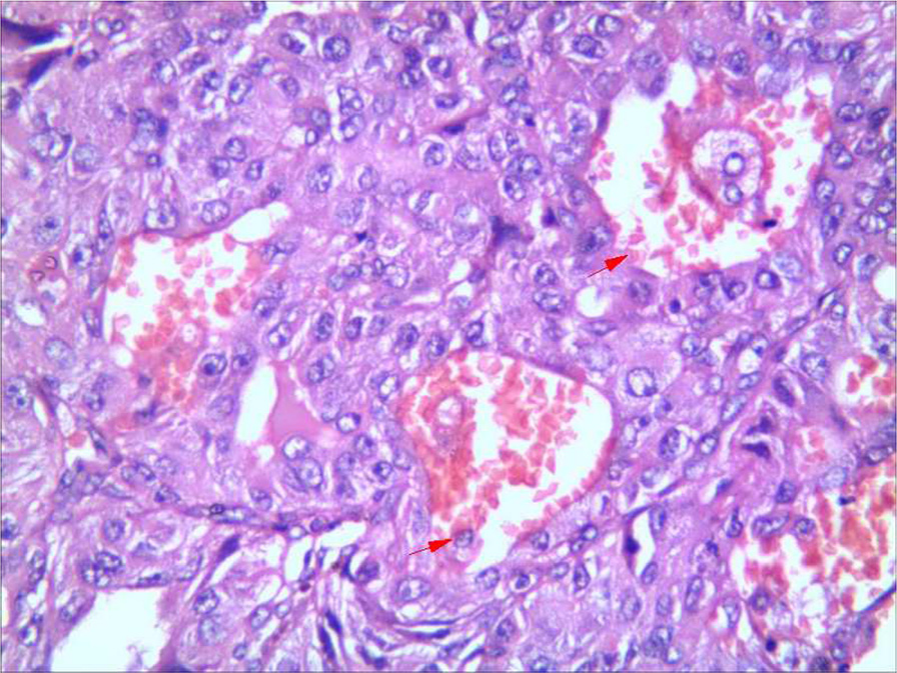
**Representative results of VM in non-small cell lung cancer and control group.** H&E staining in moderately differentiated squamous cell carcinoma, red arrow is VM (Figure 3, Figure 5 and Figure 6 are serial sections)(grade 2).

**Figure 6:**
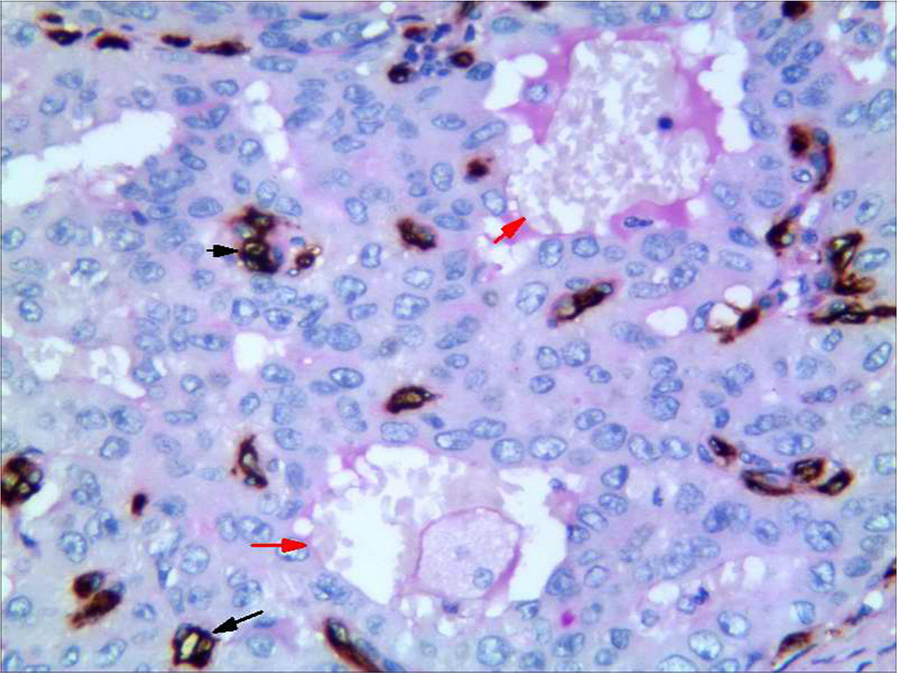
**Representative results of VM in non-small cell lung cancer and control group.** Endothelial cells are detected with anti-CD34 immunhistochemistry staning (dark brown) and vascular basement membrane with PAS staining (purple magenta) (red arrow: vessel stained positively with PAS but negatively with CD34; black arrow: vessel stained positively with CD34).

**Figure 7:**
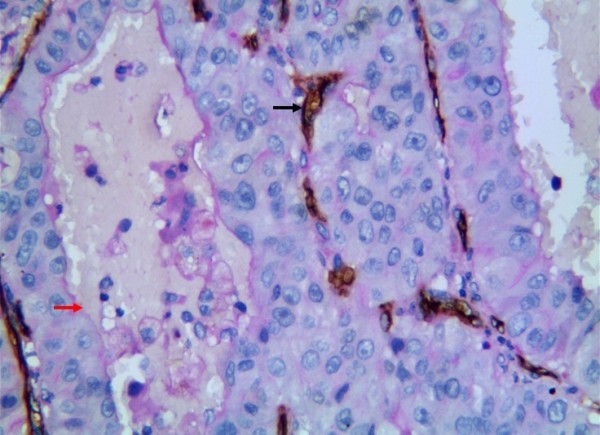
**Representative results of VM in non-small cell lung cancer and control group.** VM in moderately differentiated squamous cell carcinoma (red arrow is vessel, black arrow is VM) (grade 2) (CD34+PAS×400).

CD133 protein was expressed positively in 48.9% (149/305) of NSCLC and 26.7% (8/30) of distal normal lung tissue. The staining for CD133 was confined to the membrane and cytoplasm. There was a significant difference between the NSCLC group and the distal normal lung tissue (P<0.05) (Figures 1, 2, and 3). There was a significant difference between expression of CD133 and histological grade, pTNM stage and lymph node metastasis (P<0.001). None of the distal normal lung tissues contained VM (Figure 4). Small vessel-like structures in the tumor that were PAS-positive but CD34-negative were to be VM channels. The cells external to the lumen of the VM channels were positive for tumor cells, which indicated the channels were formed by NSCLC cells (Figure 5, 6, and 7). The presence as well as pattern (linear, tubular, and network) of VM channels were recorded in each case. VM was identified in NSCLC tissue from 109 (35.7%) of 305 specimens of NSCLC. There was a significant difference between the NSCLC group and the distal normal lung group (P<0.05) (Table 1). And the positive staining of VM and MVD score were found to be closely linked to pTNM stages, grade of tumors, lymph node metastasis in NSCLC (P<0.05). However, the positive expression of CD133, VM and MVD score were no significant association with gender, age, gross type, histological type and diameter of tumors (P>0.05) (Table 1).

**Table 1:** Correlation of CD133 expression and VM to clinicopathological characteristics in NSCLC

**Variable**	**CD133**		***P *value**	**VM**		***P *value**	**MVD**		***P *value**
	**Negative**	**Positive**		**Negative**	**Positive**		**(mean)**	**F**	
Tissue			<0.05			<0.001			
Normal	22	8		0	30				
NSCLC	156	149		196	109				
Gender			>0.05			>0.05		0.154	>0.05
Male	117	116		150	83		22.2±11.3		
Female	39	33		46	26		21.6±10.9		
Ages			>0.05			>0.05		0.476	>0.05
<60	77	64		93	48		21.5±11.8		
≥60	79	85		103	61		22.4±10.7		
Gross type			>0.05			>0.05		0.004	>0.05
Central	114	110		141	83		22.0±11.6		
Peripheral	42	39		55	26		21.9±10.1		
Histological type			>0.05			>0.05		3.550	>0.05
Squamous carcinoma	111	99		141	69		21.2±11.3		
Adenocarcinoma	45	50		55	40		23.8±10.7		
Diameter of tumor			>0.05			>0.05		0.133	>0.05
<3.0cm	13	17		20	10		21.3±11.1		
≥3.0cm	143	132		176	99		22.1±11.2		
Differentiation			<0.001			<0.001		31.231	<0.001
Well	30	4		32	2		13.6±7.1		
Moderate	104	99		142	61		20.9±9.7		
Poor	22	46		22	46		29.6±12.9		
Lymph node metastasis			<0.001			<0.001		67.650	<0.001
Yes	71	114		93	92		25.9±10.2		
No	85	35		103	17		16.1±10.0		
pTNM stage			<0.001			<0.001		200.694	<0.001
Iand II	111	29		129	11		14.3±6.7		
III and IV	45	120		67	98		28.5±10.1		
VM			<0.001					471.458	<0.001
Negative	143	53					15.5±5.9		
Positive	13	96					33.7±8.7		
CD133						<0.001		216.747	<0.001
Negative				143	13		15.0±7.2		
Positive				53	96		29.4±9.8		

### Prognosis and multivariate analysis

**Figure 8:**
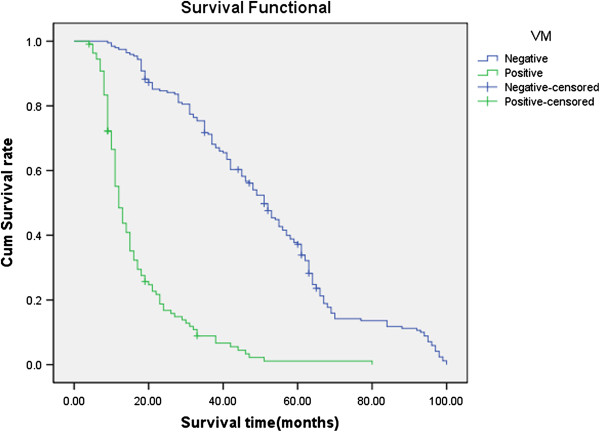
**Kaplan-Meier survival analysis by CD133, VM and MVD status (A is CD133; B is VM; C is MVD). (n=305).** The y-axis represents the percentage of patients; the x-axis, their survival in months. the green line represents CD133+ patients with a trend of worse survival than the blue line representing CD133- non-small cell lung cancer patients (P<0.001). Mean survival times were 19.0 months for the CD133+ group and 58.1 months for the CD133- group.

**Figure 9:**
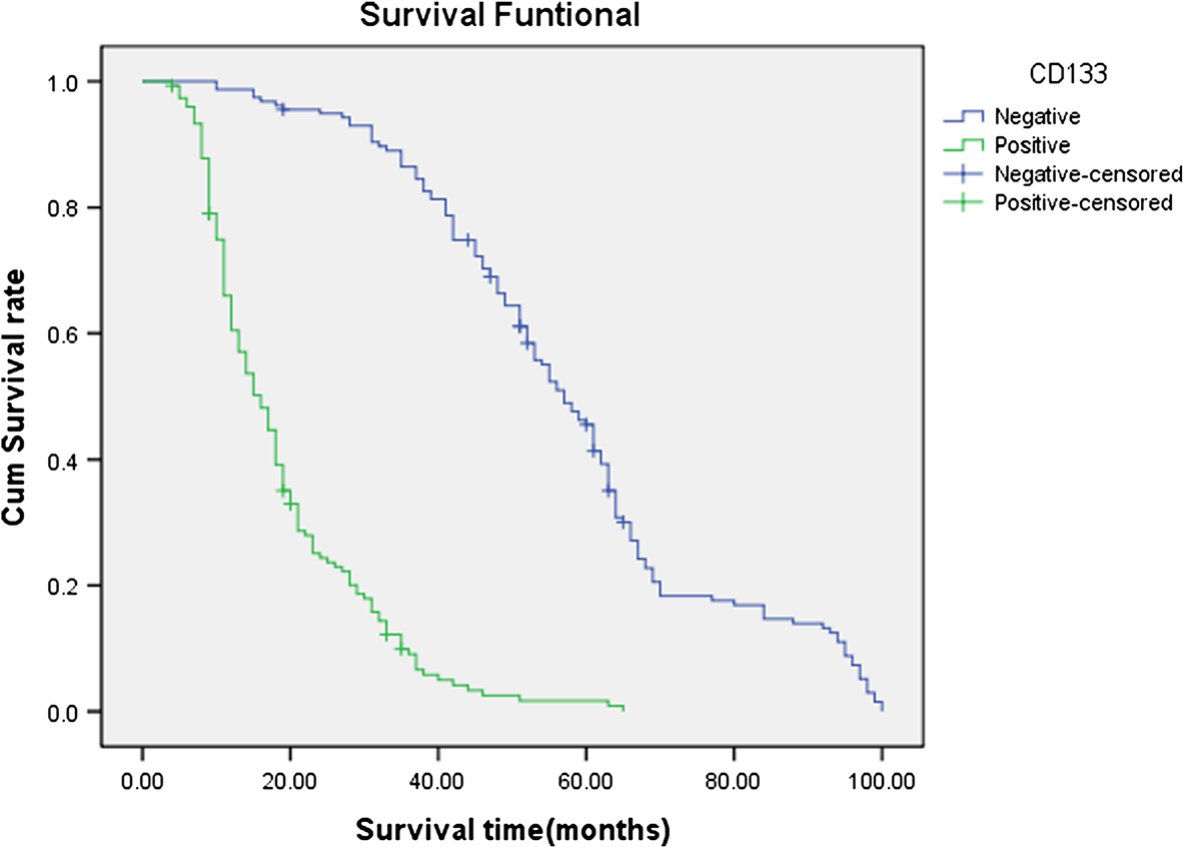
**Kaplan-Meier survival analysis by CD133, VM and MVD status (A is CD133; B is VM; C is MVD). (n=305).** The y-axis represents the percentage of patients; the x-axis, their survival in months. The green line represents VM positive patients with a trend of worse survival than the blue line representing VM negative NSCLC patients (P<0.001). Mean survival times were 16.9 months for the VM positive group and 51.4 months for the VM negative group.

**Figure 10:**
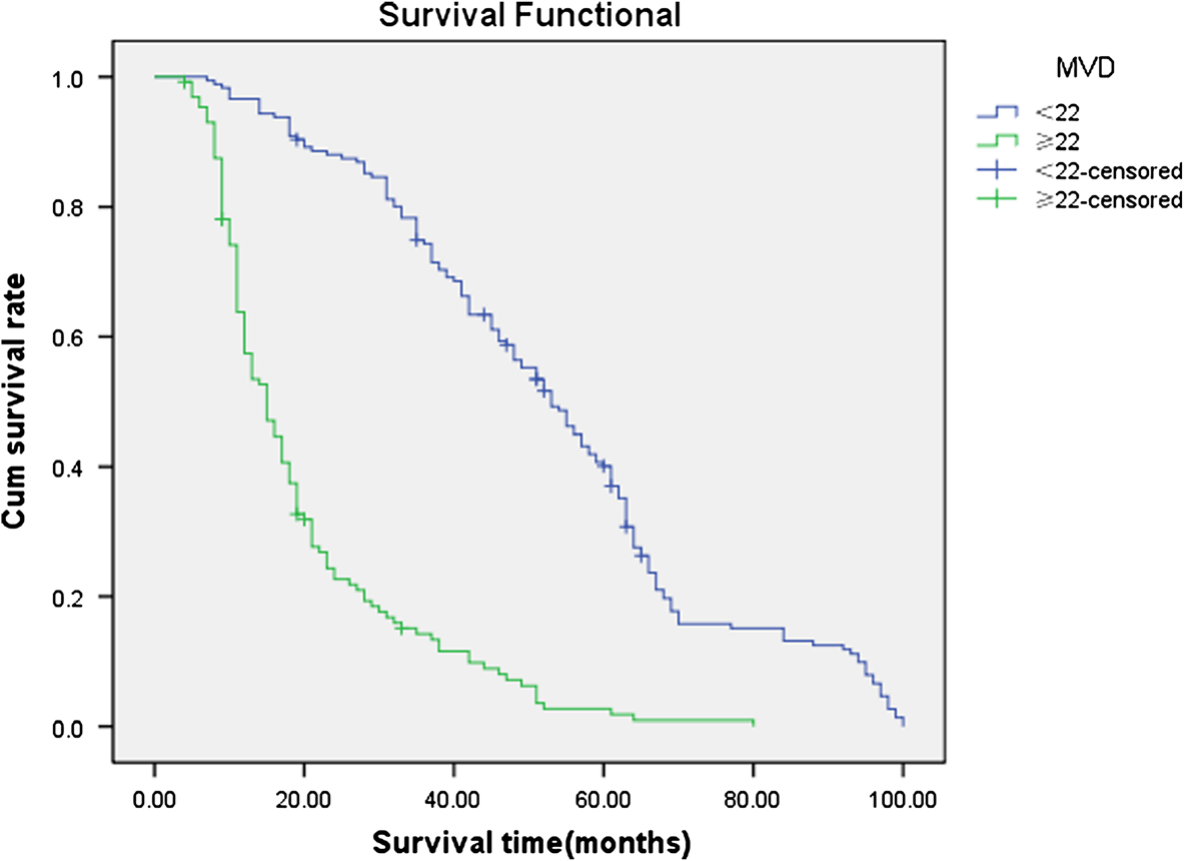
**Kaplan-Meier survival analysis by CD133, VM and MVD status (A is CD133; B is VM; C is MVD). (n=305).** The y-axis represents the percentage of patients; the x-axis, their survival in months. The green line represents MVD≥22 group(because the mean score of MVD is 22.0) with a trend of worse survival than the blue line representing MVD<22 group NSCLC patients (P<0.001). Mean survival times were 19.8 months for MVD≥22 group and 53.2 months for MVD<22 group.

Follow-up data showed that a significantly decreasing trend in the overall mean survival time between the carcinomas with the expression of CD133 (19.0 months) and those without (58.1 months)(Log rank=247.8, P < 0.001). The survival rate of the VM group was significantly less than the non-VM group (P<0.001); the survival rate of the MVD<22 group was significantly more than the MVD≥22 group (because the mean score of MVD was 22.0±11.2) (Figures 8, 9 and 10. CD133, VM, MVD, postoperative therapy and pTNM stages were independent prognostic factors by multivariate analysis (P<0.05)) (Table 2).

**Table 2:** Multivariate survival analysis of 305 patients with NSCLC

**Covariate**	**B**	**SE**	**Sig**	**Exp(B)**	**95%CI**
pTNM	0.425	0.196	0.030	1.530	1.042-2.246
VM	0.649	0.226	0.004	1.914	1.229-2.982
CD133	1.632	0.181	0.000	5.116	3.587-7.298
MVD	0.515	0.226	0.023	1.674	1.075-2.606
Therapy	0.325	0.162	0.045	0.723	0.526-0.992

### Correlation of CD133, VM and MVD in NSCLC

In positive expression of CD133 group, the mean score of MVD was 29.4±9.8; in negative expression of CD133 group, the mean score of MVD was 15.0±7.2. There was a positive correlation between CD133 and MVD (r=0.685, P<0.001), and the same correlation between VM and MVD (r=0.777, P<0.001). The positive correlation between CD133 and VM was found (r=0.585, P<0.001).

## Discussion

Cancer stem cells, also known as tumor initiating cells, are defined as a subset of tumor cells with the capacity to self-renewal and give rise to the differentiated cells that comprise the bulk of the tumor [3,5,6,9,13,45]. CD133 is commonly regarded as a marker of CSCs or TIC in many tumors [9,16-27]. Recently, CD133 has been identified in more or equal to 50% of NSCLC, colon, gastric and ovarian [9,19,22,46]. Lung cancer CD133+ cells were able to grow indefinitely as tumor spheres; upon differentiation, lung cancer CD133+ cells acquired the specific lineage markers, while loosed the tumorigenic potential together with CD133 expression [47]. Furthermore, CD133+ cancer cells were more efficient at forming clones and proliferated more extensively than the CD133- population [46]. Lung cancer stem cells are resistant to chemotherapeutic drugs, in line with the poor therapeutic effect of conventional chemotherapy on lung cancer patients [47]. Therefore, anti-CD133 gene-drug conjugates may warrant further evaluation as a molecular therapeutic strategy to eradicate CD133+ cancer cells in lung cancer. To our data, there are no reports about the prognostic significance and correlation between CD133 expression and VM in NSCLC. Using immunohistochemistry, we detect the expression of CD133 in 149 (48.9%) and it is closely related to the worst prognosis of NSCLC. Further research shows that the expression of CD133 may be involved in grade of NSCLC, lymphoid node metastasis and pTNM stages. And the positive expression of CD133 indicates a shorter survival time. Our study is consistent with parts of previous studies in lung carcinoma [48-50]. Another result is that not only normal lung tissue (bronchiolar epithelial cells) but also cancer cell expresses CD133. Although CD133, which is the most accredited, is useful stem cell markers, it is known to be expressed also in non-stem cell populations and play an important role in tumorigenesis [51]. It has been shown that CD133 is an apical molecule in many normal human tissues and its expression is not restricted to stem cells in pancreatic tissues [52]. In addition, CD133 has been taken as a prognostic factor of some cancers, such as colon carcinoma, non-small cell lung cancer, hepatocellular carcinoma and cholangiocarcinoma [48-51,53-55]. Our result is similar to the previous studies (including lung and other tissues) [22,49,52,56]. As the population of CD133+ cells in NSCLC may represent a relatively large portion of cells (up to 48.9%) and only a part of CD133+ cells possesses the abilities of stem cells [47]. These cancer stem cells after routine chemo- and radio-therapy may lead to tumor recurrence and metastasis through the self-renewal, the multiple differentiating potential and the proliferating aptitude in line with cancer stem cell hypothesis [57].

Some highly aggressive human tumor cells mimic endothelial cells and form vascular channel-like structures to convey blood and nutrients without the participation of endothelial cells. In other words, the term VM is appropriate to describe the formation of these vascular channels by tumor cells. In the H&E staining, we observe that VM channels are composed of a basement membrane with positive PAS staining and CD34- in the absence of endothelial cells. Based on PAS and CD34 staining, some morphologic patterns of VM including straight lines, arcs, loops, networks and patterns have been described [58]. In our study, VM is observed in 35.7% (109/305) of NSCLC patients. And we find that VM is associated with grade of tumor, lymph node metastasis and pTNM stages, which are in agreement with previous reports [59,60]; further research, we find that the VM-positive-group correlates with the lower survival time, suggests that the existence of VM increases the likelihood of hematogenous metastases and is in inverse proportion to prognosis [34].

Microvessel density (MVD) is the standard method of measuring tumor angiogenesis and is closely related with tumor growth and postoperative prognosis. So the patients with high scores of MVD indicate early metastasis and short survival [61,62]. Our data is consistent with these results. CD133 is not expressed in all tumors, and expressed only a small fraction of the tumor cells in CD133-positive tumors. The niche where CSCs reside may regulate CSCs self-renewal. Vascular niche may regulate CSCs fate. Alternatively, perhaps CD133 is a marker that does not initiate but rather enhances tumor growth, for example, by means of better tumor vascularization [51]. Our research also shows that CD133, VM and MVD are positively correlated with each other in CD133-positive population, suggesting that CSCs may be related to angiogenesis and vasculogenic mimicry. CSCs have self-renewal properties, multiple differentiating potential and proliferating aptitude. Tumors require a blood and nutrient supply for growth and metastasis. When the diameter of tumor is more than 1 mm3 in volume, tumor can stimulate angiogenesis in order to get adequate blood, the CD133 positive cells may contribute to the formation of capillaries [62]. But when the blood supplied by neovascularization cannot meet the needs of tumor growth, some CSCs can mimic endothelial cells and form vascular channel-like structures, which is VM [63]. The microvessel and VM sustain CSCs self-renewal, multiple differentiating potential and proliferating aptitude. The combined detection of CD133, VM and MVD, to some extent, can reflect the biological behavior of NSCLC cells, thus giving the choice of molecular targeting therapy.

## Conclusions

It is suggested that CD133 may play an important role in the evolution of NSCLC. And CD133, VM and MVD should be considered as potential marker for the prognosis in patients with NSCLC.

## Competing interests

The authors declare that they have no competing interests.

## Authors’ contributions

WSW and TSY carried out the design, analysis of pathology and drafted the manuscript. YL, ZL, CDM, and ML carried out sample collections and coordination. WDN and CZN performed the immunohistochemical staining. All authors read and approved the manuscript.

## Pre-publication history

The pre-publication history for this paper can be accessed here:

http://www.biomedcentral.com/1471-2407/12/535/prepub
